# New Nitrogen Compounds Coupled to Phenolic Units with Antioxidant and Antifungal Activities: Synthesis and Structure–Activity Relationship

**DOI:** 10.3390/molecules23102530

**Published:** 2018-10-03

**Authors:** Ana Bettencourt, Marián Castro, João Silva, Francisco Fernandes, Olga Coutinho, M. João Sousa, M. Fernanda Proença, Filipe Areias

**Affiliations:** 1 Department of Chemistry, University of Minho, Campus de Gualtar, 4710-057 Braga, Portugal; abete@quimica.uminho.pt (A.B.); francisco.fernandes@upmc.fr (F.F.); 2 Department of Pharmacology, Center for Research in Molecular Medicine and Chronic Diseases (CiMUS), Universidade de Santiago de Compostela, Avenida de Barcelona 22, 15782 Santiago de Compostela, Spain; marian.castro@usc.es; 3 Department of Biology, Universidade do Minho, Campus de Gualtar, 4710-057 Braga, Portugal; jp.silva@gmail.com (J.S.); olgapc@bio.uminho.pt (O.C.); mjsousa@bio.uminho.pt (M.J.S.); 4 School of Chemical Science and Engineering, Yachay Tech University, Yachay City of Knowledge, 100650 Urcuqui, Ecuador

**Keywords:** Imidazoles, phenolic compounds, antifungal activity, MIC, antioxidant capacity, structure-activity relationship

## Abstract

A selection of 1-amino-2-arylidenamine-1,2-(dicyano)ethenes **3** was synthesized and cyclized to 2-aryl-4,5-dicyano-1*H*-imidazoles **4** upon reflux in ethyl acetate/acetonitrile, in the presence of manganese dioxide. These compounds were tested for their antioxidant capacity by cyclic voltammetry, 2,2-diphenyl-1-picrylhydrazyl (DPPH) radical and deoxyribose degradation assays. The minimum inhibitory concentration of all compounds was evaluated against two yeast species, *Saccharomyces cerevisiae* and *Candida albicans*. Their toxicity was tested in mammal fibroblasts. Among the synthesised compounds, two presented dual antioxidant/antifungal activity without toxic effects in fibroblasts. The new compounds synthesized in this work are potential biochemical tools and/or therapeutic drugs.

## 1. Introduction

The synthesis of novel antioxidants is an important area for the pharmaceutical, cosmetic, and food industries [1,2]. Indeed, antioxidants have matchless properties in the conservation of many market products, and an important function in the prevention of many degenerative pathologies, such as, cancer, cardiovascular diseases, arthritis, arteriosclerosis, and the normal process of aging [3,4].

The antioxidant capacity of a compound is associated with its ability to act as a scavenger of free radicals [5]. The chelation capacity of metal ions is another important property of an efficient antioxidant, because iron and copper ions can initiate the production of hydroxyl radicals by the Fenton reaction [6]. Phenolic compounds, such as quercetin and gallic acid, are typical examples of potent antioxidants [7].

The resistance of pathogenic fungi to actual drugs on the market supports the need for the synthesis of novel antifungal agents [8], presenting selective toxicity to fungi without toxic effects to other organisms [9]. Nevertheless, commercial antifungals do not present this selective toxicity. Additionally, the actual antifungal agents are highly persistent in the environment, and they present a threat to living beings in general [10]. Nitrogen heterocycles, like fluconazole and miconazole, are examples of systemic antifungals with a wide spectrum of action on fungi [11].

In this study, the imidazole ring, present in many antifungal agents, was coupled with the phenolic unit, which is known to confer antioxidant capacity, to create new nitrogen heterocycles. The compounds synthesized were assayed for their antioxidant and antifungal activities, and the results allowed for an understanding of a relationship between chemical structure and activity (SAR).

## 2. Results

### 2.1. Chemistry

The products and reactions reported in this section are summarized in Table 1. The 1,2-(dicyano)ethenes **3a**–**3e**, used as starting materials for the imidazole scaffolds studied in this work, were prepared by the reaction of diaminomaleonitrilo **1** with phenolic aldehydes **2a**–**2e**, in ethanol and using sulphuric acid as catalyst. The products **3a**–**3e** were isolated at 81–97% yield after approximately 10 minutes at room temperature.

The cyclization of the 1,2-(dicyano)ethenes **3a**–**3e** to the corresponding imidazoles **4a**–**4d** was performed under reflux in tetrahydrofuran (THF), using an excess of MnO_2_. The products were isolated in 50–70% yield after 8 hr to 5 days. All attempts to prepare compound **4e** from **3e**, with either acid or base catalysis, varying the solvent and using nitrogen atmosphere, resulted in the formation of dark materials, possibly as the result of extensive oxidation.

### 2.2. Antioxidant Capacity

The antioxidant capacity of 1,2-(dicyano)ethenes **3a**–**3e** and imidazoles **4a**–**4d** was assessed through a step-by-step cascade using three different experimental procedures. Initially, we determined the anodic peak potential (E_p_) of all compounds by the cyclic voltammetry technique because this potential represents the ability of compounds to donate their electrons, and it can be used as a parameter of the scavenging activity [12]. Subsequently, we determined and confirmed the scavenging activity by the DPPH^●^ assay for the compounds that presented the lowest potentials of each chemical series (**3e** and **4d**) [13]. Finally, we determined the antioxidant capacity by the deoxyribose degradation assay for the two previous compounds, because this assay can indicate both the scavenging and the chelating activities of these compounds [14]. The results obtained were compared with the reference antioxidant trolox.

Previous work showed that compounds selected by their electrochemical behavior are likely to act as radical scavengers [12]. In this study, cyclic voltammetry was used to test the anodic peak potential of the synthesized compounds. The anodic peak potential (E_p_) and the half peak potential (E_p/2_) were used to measure the easiness of oxidation, and consequently the antioxidant capacity of the compounds. The obtained peak and half peak potentials are presented in Table 2. The oxidation peak potentials of the tested compounds vary from 0.113 V up to 0.896 vs. saturated calomel electrode (SCE), depending on the number of hydroxyl groups in the aromatic ring. Published literature corroborates our current observation, stating that compounds with two or more hydroxyl groups have lower anodic peak potentials and higher antioxidant capacities than monosubstituted phenols [15]. The voltammograms obtained for the oxidation of 1,2-(dicyano)ethenes **3a**–**3c** with the 2-hydroxy, 3-hydroxy, and 4-hydroxy substituents in the aromatic ring presented values for the peak potential that were equal to or higher than 0.476 V. The 1,2-(dicyano)ethenes **3d**–**3e**, with the 3,4-di-hydroxyphenyl and 3,4,5-tri-hydroxyphenyl substituents presented lower peak potentials, varying from 0.113 to 0.248 V. These differences in electrochemical behavior indicate that the number of hydroxyl groups of the phenolic unit is responsible for the easiness of oxidation of the compounds.

The peak potentials of compounds **3d**–**3e** were of the same order of magnitude or even lower than the peak potential of trolox (E_p_ = 0.173 V), suggesting that these compounds have a high scavenging capacity. Indeed, the peak potentials of the previous compounds (**3d**–**3e**) were compared with the peak potential for trolox, a standard antioxidant, as these values represented the tendency of the compounds to give up their electrons. In the literature, antioxidant/prooxidant activity has been associated with the oxidation potential, and compounds with a low peak potential (lower than 0.450 mV) were identified as antioxidants [12,15]. 

For each compound series, the scavenging activity of the synthesized molecules with the lowest peak potential (**3e** and **4d**) was assessed by the DPPH radical assay. Compound **3e** (3.7 ± 0.7 µM) presented an IC_50_ value that was lower than trolox (9.0 ± 0.2 µM), establishing that this compound is an efficient radical scavenger. 

The imidazole **4d**, substituted with the 3,4-di-hydroxyphenyl moiety, presented a peak potential value of 0.254 V, which was the lowest of this series (Table 2). The IC_50_ value obtained for this imidazole in the DPPH radical assay, 12.0 ± 1.0 µM, showed that there was a slight decrease in the scavenging activity as consequence of cyclization. Despite this decrease in activity, imidazole **4d** could be considered to be a scavenger that is almost as potent as trolox.

To finalize the antioxidant study, the most potent scavengers of each chemical series were assayed on the deoxyribose degradation assay. The compounds were assayed at their IC_50_ concentration, as determined by the DPPH^●^ assay. This experimental approach permitted us to estimate the antioxidant capacity of the synthesized compounds at equipotent scavenging concentrations. The compounds **3e** and **4d** inhibited the deoxyribose degradation by approximately 60%, whereas trolox inhibited deoxyribose degradation only by 23.4 ± 2.6%. The higher antioxidant capacity of the compounds assessed, substituted with 3,4-di-hydroxyphenyl or 3,4,5-tri-hydroxyphenyl moieties, would be a consequence of the combination of their scavenging activity and of their capacity to chelate iron ions. These results agree with the literature reporting that phenolic compounds with 3,4-di-hydroxyphenyl or 3,4,5-tri-hydroxyphenyl moieties, such as quercetin and gallic acid, respectively, have potent scavenging activities and chelating capacities for iron, and both contribute to their high antioxidant activities [16,17,18,19]. Our hypothesis was valid because the deoxyribose was oxidized with hydroxyl radicals (HO^●^) produced through the ascorbate–iron pair by the Fenton reaction [14]. These results are important, as many pathological diseases are associated with an increase of free iron concentration, as a consequence of a disruption of iron homeostasis [20]. Additionally, free iron can react with ascorbate that exists at high concentration inside the cells, generating HO^●^ by the Fenton reaction [21]. In summary, compounds **3e** and **4d** present excellent properties as antioxidant agents.

### 2.3. Antifungal Activity

The antifungal activity of the synthesized compounds was tested on two yeast species by the method of liquid microdilution, and compared with the reference antifungals fluconazole and miconazole. We chose the *Saccharomyces cerevisiae* as the model yeast in this study, because it is not pathogenic at normal conditions, but it can provoke serious infections in persons with a depressed immune system, such as patients with human immunodeficiency virus infection and acquired immune deficiency syndrome (HIV/AIDS) [22]. We also tested the activity of the compounds on *Candida albicans* because it is an opportunistic yeast that is frequently found in systemic infections [23]. Therefore, there is a high interest in the development of new antifungals with activity on these two yeasts.

Minimum inhibitory concentration (MIC) values, defined as the lowest concentration of compound able to cause 80% inhibition of cellular growth, were assessed for all compounds. Miconazole presented MIC values on *Saccharomyces cerevisiae* and *Candida albicans* of 100.0 and 0.78 µM, respectively, while MIC values for fluconazole were 50.0 and 1.56 µM, respectively (Table 3). These MIC values exhibited showed that miconazole is more potent than fluconazole on *Candida albicans*, while fluconazole is more potent than miconazole on *Saccharomyces cerevisiae*. In addition, *Candida albicans* is more sensitive than *Saccharomyces cerevisiae* to these two antifungal agents. 

In the assessment of the antifungal activity of 1,2-(dicyano)ethenes **3a**–**3e**, we observed that compound **3e**, with a 3,4,5-tri-hydroxyphenyl moiety, achieved 80% inhibition of the growth of *Saccharomyces cerevisiae* at a concentration of 50 µM (Table 3). Therefore, **3e** showed antifungal activity that was equipotent to fluconazole, and more potent than miconazole. On *Candida albicans*, **3e** presented a MIC of 100 µM, having weaker activity than miconazole and fluconazole. The highest antifungal activity of **3e** compared to the other compounds of this series seemed to be related with the lowest anodic peak potential and the highest antioxidant capacity of this compound. Therefore, we confirmed the presence of a synergistic effect between the phenolic moiety (3,4,5-tri-hydroxyphenyl) and the rest of the molecule common to all 1,2-(dicyano)ethenes. The other compounds of the series did not achieve the MIC on the yeast species considered in our study at the highest concentration evaluated in each case, due to their limited solubility in aqueous solution (Table 3).

In the evaluation of antifungal activity of imidazoles **4a**–**4d**, we observed that **4d** with the 3,4-di-hydroxyphenyl unit had MIC values of 400 and 600 µM on *Saccharomyces cerevisiae* and *Candida albicans*, respectively, showing weaker activity than fluconazole and miconazole. The highest antifungal activity of **4d** appeared to be also related with its highest antioxidant capacity in this series. There was also an increase of antifungal activity with the cyclization, because the 1,2-(dicyano)ethene **3d**, which is the respective linear precursor of imidazole **4d**, did not present any antifungal activity.

These results agree with our initial hypothesis, because there is a synergistic effect when the phenolic moiety is coupled to the nitrogen heterocycle, which was based on previous published works demonstrating that the antifungal activity increases when a phenolic antioxidant is added to a solution of antifungal agent [24]. Nevertheless, market antifungal agents do not incorporate a phenolic moiety, but they are coupled to aromatic groups substituted with chlorine or fluorine atoms [25]. Therefore, these market antifungal agents have low solubility in water and adverse consequences to the environment [10].

As previously explained, an ideal antifungal must be highly toxic for fungi and not present any toxicity for other living beings [26]. Therefore, we studied the toxicity of **3e** and **4d** on fibroblasts using the lactate dehydrogenase (LDH) release method to obtain toxicological information in mammalian cells [27]. We observed that these two compounds were not toxic for fibroblasts at concentrations that presented antifungal activity, because there was no significant release of LDH to the extracellular media (Table 3). The results showed that **3e** and **4d** presented antifungal activity at concentrations that did not result in toxicity for fibroblasts. In addition, the two compounds have the advantage of being substituted with phenolic units, which form hydrogen bonds with water molecules, and as a result, they have more solubility in the aqueous medium than compounds substituted with chlorine and fluorine atoms as the market antifungal agents. 

## 3. Materials and Methods 

### 3.1. Chemistry

Chemicals and solvents were of analytical grade and they were purchased from market sources and used without purification.

All compounds were fully characterized by spectroscopic data and compared with the data available in the literature. The IR spectra were recorded on a Bomem MB 104 instrument (ABB Bomem Inc., Quebec, Canada) using sodium chloride plates and Nujol mulls. The NMR spectra were recorded on a Varian Unity Plus (^1^H: 300 MHz and ^13^C: 75 MHz) or on a Bruker Avance 3400 (^1^H: 400 MHz and ^13^C: 100 MHz) (Bruker Corporation, Billerica, MA, USA), and deuterated DMSO was used as solvent. The chemical shifts were expressed in parts per million (ppm), and the coupling constants, J, were reported in Hertz (Hz). The peak patterns were explained as follows: s, singlet; d, doublet; t, triplet; m, multiplet. The reactions were monitored by thin layer chromatography (TLC) using aluminium plates pre-coated with silica gel 60 F254 (Merck) and mixtures of dichloromethane/ethanol (9/1) and/or chloroform/ethyl acetate/ acetic acid (16/8/1) as an eluent. The melting points were determined on a Stuart SMP3 melting point apparatus.

#### 3.1.1. General Procedure for the Synthesis of the 1-amino-2-arylidenamine-1,2-(dicyano)ethenes (**3a**–**3e**)

One drop of concentrated sulphuric acid (0.01 mL) was added to a suspension of diaminomaleonitrile **1** and benzaldehyde **2** (1 molar equivalent) in ethanol. The reaction was stirred for 10 minutes at room temperature. The yellow solid was filtered, washed with cold diethyl ether, and identified as the title compound **3**.

*1-Amino-1,2-dicyano-2-(2′-hydroxybenzylidenamine) ethene* (**3a**). 1 (0.65 g, 6.0 mmol), 2-hydroxibenzaldehyde 2a (0.65 mL, 6.08 mmol), ethanol (10 mL). Yellow solid identified as **3a** (1.24 g, 97%). Mp 251–254 °C. IR (NaCl): 3465, 3404, 3343, 3191, 2244, 2207, 1626, 1615, 1608, 1562, 1496, 1279, 1230, 1213, 1157, 1118. ^1^H-NMR (DMSO-*d*_6_, 300 MHz) δ 6.88 (t, 1H, *J* = 7.8 Hz, H*′*_5_); 6.92 (dd, 1H, *J*_1_ = 8.1 Hz, J_2_ = 0.6 Hz, H*′*_3_), 7.32 (dt, 1H, *J*_1_ = 7.8 Hz, *J*_2_ = 1.8 Hz, H*′*_4_), 8.03 (dd, 1H, *J*_1_ = 7.8 Hz, *J*_2_ = 1.8 Hz, H*′*_6_), 8.58 (s, 1H, H_4_), 7.84 (s, 2H, NH_2_), 10.42 (s, 1H, OH*′*). ^13^C-NMR (DMSO-*d*_6_, 300 MHz) δ 158,2 (C*′*_2_), 152.8 (C_4_), 133.2 (C*′*_4_), 128.9 (C*′*_6_), 126.1 (C_1_), 121.2 (C*′*_1_), 119.4 (C′_5_), 116.4 (C′_3_), 114.6 (CN), 113.4 (CN), 103.4 (C_2_). Anal. Calcd. for C_11_H_8_N_4_O: C, 62.3; H, 3.8; N, 26.4. Found: C, 62.3; H, 3.8; N, 26.1. 

*1-Amino-1,2-dicyano-2-(3´-hydroxibenzilidenamine) ethene* (**3b**). 1 (0.65 g, 6.0 mmol), 3-hydroxybenzaldehyde 2b (0.73 g, 6.0 mmol), ethanol (8 mL). Yellow solid identified as **3b** (1.04 g, 82%). Mp 231–233 °C. IR (NaCl): 3401, 3379, 3299, 3205, 2244, 2205, 1625, 1611, 1587, 1561, 1491, 1396, 1288, 1225, 1215, 1150. ^1^H-NMR (DMSO-*d*_6_, 300 MHz) δ 6.90 (ddd, 1H, *J*_1_ = 8.1 Hz, *J*_2_ = 2.4, *J*_3_ = 0.9 Hz, H′_4_), 7.25 (t, *J* = 8.1 Hz,1H, H′_5_), 7.37 (t, 1H, *J* = 2.1, H′_2_), 7.43 (d, 1H, *J* = 8.1 Hz, H′_6_), 7.88 (s, 2H, NH_2_), 8.16 (s, 1H, H_4_), 9.64 (s, 1H, OH′). ^13^C-NMR (DMSO-*d*_6_, 300 MHz) δ 157.7 (C′_3_), 155.4 (C_4_), 136.8 (C′_1_), 129.7 (C′_5_), 126.8 (C_1_), 120.3 (C′_6_), 118.8 (C′_4_), 115.3 (C′_2_), 114.5 (CN), 113.8 (CN), 102.7 (C_2_). Anal. Calcd. for C_11_H_8_N_4_O: C, 62.3; H, 3.8; N, 26.4. Found: C, 62.3; H, 3.9; N, 26.3.

*1-Amino-1,2-dicyano-2-(4´-hydroxybenzilidenamine) ethene* (**3c**). 1 (0.65 g, 6.0 mmol), 4-hydroxybenzaldehyde **2c** (0.73 g, 6.0 mmol), ethanol (10 mL). Yellow solid identified as **3c** (1.11 g, 87%). Mp 226–228 °C. IR (NaCl): 3455, 3410, 3322, 3272, 3184, 2231, 2207, 1625, 1610, 1589, 1567, 1512, 1306, 1264, 1235, 1207, 1162, 1112. ^1^H-NMR (DMSO-*d*_6_, 300 MHz) δ 6.82 (d, 2H, *J* = 8.7 Hz, H′_3+5_), 7.65(s, 2H, NH_2_), 7.86 (d, 2H, *J* = 8.7 Hz, H′_2+6_), 8.14 (s, 1H, H_4_), 10.21 (s, 1H, OH′). ^13^C-NMR (DMSO-*d*_6_, 300 MHz) δ 161.0 (C′*_p_*), 155.1 (C_4_), 131.2 (C′*_o_*), 126.9 (C′_1_), 125.5 (C_1_), 115.7 (C′*_m_*), 114.7 (CN), 114.0 (CN), 103.4 (C_2_). Anal. Calcd. for C_11_H_8_N_4_O.1,1H_2_O: C, 57.0; H, 4.4; N, 24.2. Found: C, 57.2; H, 4.4; N, 24.0.

*1-Amine-1,2-dicyano-2-(3´,4´-dihydroxybenzilidenamine) ethene* (**3d**). 1 (0.32 g, 3.0 mmol), 3,4-dihydroxybenzaldehyde 2d (0.41 g, 3.0 mmol), ethanol (1.5 mL). Yellow solid identified as **3d** (0.55 g, 81%). Mp 219–222 °C. IR (NaCl): 3465, 3349, 3297, 3169, 2230, 2201, 1603, 1590, 1565, 1528, 1340, 1299, 1264, 1193, 1167, 1102. ^1^H-NMR (DMSO-*d*_6_, 300 MHz) δ 6.81 (d, 1H, *J* = 8.1, H′_5_), 7.29 (dd, 1H, *J*_1_ = 8.1 Hz, J_2_ = 2.0 Hz, H′_6_), 7.40 (d, 1H, *J* = 2.0 Hz, H′_2_), 7.60 (s, 2H, NH_2_), 8.06 (s, 1H, H_4_), 9.15 (s, 1H, OH′), 9.80 (s, 1H, OH′). ^13^C-NMR (DMSO-*d*_6_, 300 MHz) δ 155.6 (C_4_), 149.7 (C′_4_), 145.7 (C′_3_), 127.4 (C′_1_), 125.3 (C_1_), 122.8 (C′_6_), 115.5 (C′_2_), 115.5 (C′_5_), 114.8 (CN), 114.0 (CN), 103.6 (C_2_). Anal. Calcd. for C_11_H_8_N_4_O_2_.1,1H_2_O: C, 53,5; H, 4.1; N, 22.7. Found: C, 53.5; H, 4.1; N, 22.7.

*1-Amino-1,2-dicyano-2-(3´,4´,5´-trihydroxybenzilidenamine)ethene* (**3e**). 1 (0.22 g, 2.0 mmol), 3,4,5-trihydroxybenzaldehyde 2e (0.34 g, 2.0 mmol), ethanol (1 mL). Yellow solid identified as **3e** (0.45 g, 92%). Mp > 300 °C (dec.). IR (NaCl): 3473, 3445, 3359, 3256, 3182, 2242, 2205, 1606, 1591, 1561, 1529, 1401, 1338, 1311, 1231, 1190, 1177, 1136. ^1^H-NMR (DMSO-*d*_6_, 300 MHz) δ 6.93 (s, 2H, H′_2+6_), 7.56 (s, 2H, NH_2_), 7.98 (s, 1H, H_4_), 9.09 (s, >2H; OH′). ^13^C-NMR (DMSO-*d*_6_, 300 MHz) δ 156.1 (C_4_), 146.1 (C′*_m_*), 138.0 (C′*_p_*), 126.1 (C′_1_), 125.1 (C_1_), 114.8 (CN), 114.1 (CN), 108.7 (C′*_o_*), 103.7 (C_2_). Anal. Calcd. for C_11_H_8_N_4_O_3_.0,1H_2_O: C, 53.7; H, 3.4; N, 22.8. Found: C, 53.7; H, 3.5; N, 22.6.

#### 3.1.2. General Procedure for the Synthesis of the 2-aryl-4,5-dicyano-1H-imidazoles (**4a**–**4d**)

Manganese dioxide was added to a solution of **3** in ethyl acetate/acetonitrile, and the mixture was kept under reflux until TLC indicated the complete consumption of reagent **3**. The manganese dioxide suspension was filtered using fiberglass paper, and the solvent was partially removed under reduced pressure. The yellow solid was filtered, washed with cold dichloromethane, and identified as the title compound **4**.

*4,5-Dicyano-2-(2´-hydroxyphenyl)-1H-imidazole* (**4a**). 3a (0.30 g, 1.42 mmol), manganese dioxide (6.16 g), ethyl acetate/acetonitrile (50/5 mL) and reflux for 14 hours. The yellow solid was identified as **4a** (0.15 g, 50%). Mp > 300 °C (dec.). IR (NaCl): 3441, 3335, 3240, 2250, 2237, 1675, 1607, 1551, 1521, 1473, 1403, 1351, 1308, 1293, 1264, 1226, 1154, 1111. ^1^H-NMR (DMSO-*d*_6_, 300 MHz) δ 6.94 (t, 1H, *J* = 7.8 Hz, H′_5_), 7.02 (d, 1H, *J* = 7.8 Hz, H′_3_), 7.34 (dt, 1H, *J*_1_ = 7.8 Hz, *J*_2_ = 1.5 Hz, H′_4_), 7.94 (dd, 1H, *J*_1_ = 7.8 Hz, *J*_2_ = 1.5 Hz, H′_6_), 10.00–13.00 (s, >1H, NH + OH′). ^13^C-NMR (DMSO-*d*_6_, 300 MHz) δ 155.4 (C′_2_), 149.3 (C_2_), 132.1 (C′_4_), 128.7 (C′_6_), 119.7 (C′_5_), 116.5 (C′_3_), 113.8 (C′_1_), 111.5 (CN), not visible (C_4_/C_5_). Anal. Calcd. for C_11_H_6_N_4_O.0,9H_2_O: C, 58.3; H, 3.5; N, 24.7. Found: C, 58.6; H, 3.6; N, 24.4.

*4,5-Diciano-2-(3´-hidroxifenil)-1H-imidazole* (**4b**). 3b (0.30 g, 1.42 mmol), manganese dioxide (6.16 g) ethyl acetate/acetonitrile (50/5 mL) and reflux for five days. The yellow solid was identified as **4b** (0.21 g, 70%). Mp 264–267 °C. IR (NaCl): 3471, 3369, 3223, 2248, 2242, 1664, 1612, 1590, 1567, 1529, 1490, 1409, 1392, 1328, 1287, 1229, 1207, 1107. ^1^H NMR (DMSO-d_6_, 300 MHz) δ 6.91 (ddd, 1H, *J*_1_ = 7.8 Hz, *J*_2_ 2.4 Hz, *J*_3_ = 1.2 Hz, H′_4_), 7.33 (t, 1H, J = 7.8 Hz, H′_5_), 7.35-7.50 (m, 2H, H′_2+6_), 9.86 (s, 1H, OH′), 12.00–16.00 (s, <1H, NH). ^13^C-NMR (DMSO-*d*_6_, 300 MHz) δ 157.9 (C′_3_), 151.1 (C_2_), 130.4 (C′_5_), 128.8 (C′_1_), 118.0 (C′_4_), 117.0 (C′_6_), 113.0 (C′_2_), 111.4 (CN), not visible (C_4_/C_5_). Anal. Calcd. for C_11_H_6_N_4_O.0,6H_2_O: C, 59.6; H, 3.3; N, 25.3. Found: C, 59.8; H, 3.5; N, 25.0. 

*4,5-Dicyano-2-(4´-hydroxyphenyl)-1H-imidazole* (**4c**). 3c (0.30 g, 1.42 mmol), manganese dioxide (6.16 g), ethyl acetate/acetonitrile (30/5 mL) and reflux for eight days. The yellow solid was identified as **4c** (0.19 g, 63%). Mp 279–281 °C. IR (NaCl): 3435, 3387, 3148, 2242, 2233, 1648, 1613, 1593, 1492, 1403, 1311, 1281, 1241, 1177, 1123. ^1^H-NMR (DMSO-*d*_6_, 300 MHz) δ 6.89 (d, 2H, *J* = 9.0 Hz, H’_3+5_), 7.80 (d, 2H, *J* = 9.0 Hz, H’_2+6_), 10.16 (s, 1H, OH’), 12.00–16.00 (s, <1H, NH). ^13^C-NMR (DMSO-*d*_6_, 300 MHz) δ 159.9 (C′*_p_*), 151.5 (C_2_), 128.2 (C′*_o_*), 118.6 (C′_1_), 116.0 (C′*_m_*), 111.6 (CN), not visible (C_4_/C_5_). Anal. Calcd. for C_11_H_6_N_4_O.1,9H_2_O: C, 54.1; H, 4.0; N, 22.9. Found: C, 54.0; H, 4.2; N, 22.8.

*4,5-Dicyano-2-(3´,4´-dihydroxyphenyl)-1H-imidazole* (**4d**). 3d (0.25 g, 1.10 mmol), manganese dioxide (2.38 g), ethyl acetate/acetonitrile (50/10 mL) and reflux for 24 hours. The yellow solid was identified as **4d** (0.14 g, 55%). Mp > 300 °C (dec.). IR (NaCl): 3484, 3366, 3183, 2245, 2236, 1656, 1620, 1605, 1543, 1506, 1397, 1323, 1289, 1256, 1227, 1183, 1118. ^1^H-NMR (DMSO-*d*_6_, 300 MHz) δ 6.84 (d, 1H, *J* = 8.1 Hz, H′_5_), 7.28 (dd, 1H, *J*_1_ = 8.1 Hz, *J*_2_ = 2.1 Hz, H′_6_), 7.38 (d, 1H, *J* = 2.1 Hz, H′_2_), 9.38 (s, 1H, OH′), 9.63 (s, 1H, OH′) and the NH signal is not visible on spectrum. ^13^C-NMR (DMSO-*d*_6_, 300 MHz) δ 151.8 (C_2_), 148.3 (C′_4_), 145.7 (C′_3_), 119.1 (C′_1_), 118.3 (C′_6_), 116.0 (C′_5_), 113.7 (C′_2_), 111.7 (CN), not visible (C_4_/C_5_). Anal. Calcd. for C_11_H_6_N_4_O_2_.1,2H_2_O: C, 53.2; H, 3.4; N, 22.6. Found: C, 53.2; H, 3.4; N, 22.5.

### 3.2. Antioxidant Capacity 

#### 3.2.1. Cyclic Voltammetry Technique

Cyclic voltammograms were obtained in a conventional three-electrode cell. The working electrode was a glassy carbon electrode; a saturated calomel electrode (SCE) and a platinum wire were used as reference electrode and counter electrode, respectively. Cyclic voltammograms were recorded at a scan rate of 50 mV s^-1^ and all solutions were purged with argon before assays.

Solutions of the test compounds (1.0 mM) in a 1:1 (v/v) water/ethanol solution containing 0.14 M KCl and phosphate buffer (1.8 mM KH_2_PO_4_ and 10.1 mM Na_2_HPO_4_) were prepared, and the pH of the solutions was adjusted to pH = 7.4.

The experiments were done using Autolab PGSTAT 30 running with GPES (General Purpose Electrochemical System) version 4.6 software PG (Ecochimie, Utrecht, Netherlands).

#### 3.2.2. DPPH Radical Method

The DPPH^•^ scavenging activity of the compounds was measured as already described [13]. A 0.002% ethanolic DPPH solution was used in the assays, and trolox was used as reference antioxidant. The IC_50_ value was determined for the compounds with the lowest oxidation potential from each chemical series. 

#### 3.2.3. Deoxyribose Degradation Method 

Antioxidant effects of compounds on hydroxyl radicals (HO^●^) were determined as described previously [14]. Reaction mixtures contained deoxyribose, KH_2_PO_4_–KOH buffer, ascorbic acid, FeCl_3_, H_2_O_2_, and synthesized compounds (assessed at IC_50_ concentration obtained in DPPH^●^ assay). After one hour of incubation at 37 °C, the colour appeared by adding 1 mL of thiobarbituric acid and 1 mL of trichloroacetic acid. The reaction mixture was then heated in boiling water for 15 min, and the absorbance of the resulting solutions was measured at 532 nm. 

### 3.3. Determination of Antifungal Activity

MIC values were evaluated by the method of liquid microdilution described in the standard document CLSI M27-3A [28,29]. The antifungal activity of the synthesized compounds was evaluated for the yeasts *Saccharomyces cerevisiae* PYCC 4072 and *Candida albicans* PYCC 3436^T^. For all tests, a culture of yeasts was prepared in agar tubes with medium YEPD and agar to guaranty purity and an excellent growth. An inoculum was transferred to a culture medium RPMI-1640, with 0.165 M of buffer 4-morpholinepropanesulfonic acid (Merck, Darmstadt, Germany), pH = 7.0. An aliquot of 0.1 mL of this cellular suspension was added to 0.1 mL of solution with the synthesized compound in microplate wells, with the final density of the inoculum being 2.25 × 10^3^ cells/mL. The inoculated microplaques were incubated for 48 hours at temperatures of 30 and 37 °C for *Saccharomyces cerevisiae* and *Candida albicans*, respectively. The MIC values were then measured at 640 nm. Fluconazole and miconazole (Pfizer Inc., New York, NY, USA) were used as the reference controls.

### 3.4. Analysis of Toxicity in Mammalian Fibroblast

Cellular viability was determined by the lactate dehydrogenase (LDH) method, as already described [27]. The fibroblast cell line L929 (European Collection of Animal Cell Cultures) was used in the tests. Cells were incubated with the compounds and a small volume of medium was collected for the determination of extracellular LDH. The cells were after lysed to obtain the intracellular LDH. The enzyme activity was evaluated at 340 nm. The LDH released to the extracellular medium was described as a percentage of the total LDH activity. 

## 4. Conclusions

Compounds **3e** and **4d** substituted with 3,4-di-hydroxyphenyl or 3,4,5-tri-hydroxyphenyl units presented high antioxidant activities, as determined by cyclic voltammetry, DPPH^●^, and deoxyribose degradation assays. These two compounds also displayed antifungal activity against the yeasts *Saccharomyces cerevisiae* and *Candida albicans* at concentrations not toxic to fibroblasts.

The cyclization of 1,2-(dicyano)ethene **3d** producing imidazole **4d**, increased the antifungal activity. In addition, we observed a synergistic effect between the nitrogen heterocycle and the phenolic unit, combined in the same molecule. In this work, we describe the synthesis of compounds **3e** and **4d** with dual antioxidant/antifungal activity involving two original scaffolds.

## Figures and Tables

**Table 1:**
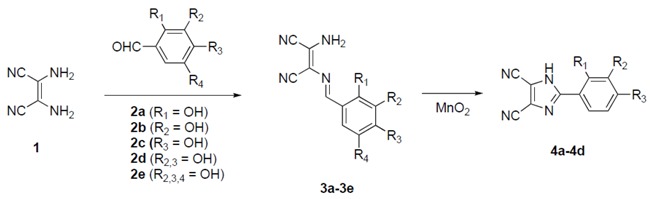
Experimental conditions for the synthesis of target compounds **3** and **4**.

Compound	R^1^	R^2^	R^3^	R^4^	Reaction Conditions	Yield (%)
**3a**	OH	H	H	H	**1** + **2a** (1.0 equiv.), EtOH, H_2_SO_4_, r.t., 10 min	97
**3b**	H	OH	H	H	**1** + **2b** (1.0 equiv.), EtOH, H_2_SO_4_, r.t., 10 min	82
**3c**	H	H	OH	H	**1** + **2c** (1.0 equiv.), EtOH, H_2_SO_4_, r.t., 10 min	87
**3d**	H	OH	OH	H	**1** + **2d** (1.0 equiv.), EtOH, H_2_SO_4_, r.t., 10 min	81
**3e**	H	OH	OH	OH	**1** + **2e** (1.0 equiv.), EtOH, H_2_SO_4_, r.t., 10 min	92
**4a**	OH	H	H	H	**3a**, ethyl acetate/acetonitrile, MnO_2_, reflux, 14 h	50
**4b**	H	OH	H	H	**3b**, ethyl acetate/acetonitrile, MnO_2_, reflux, 5 days	70
**4c**	H	H	OH	H	**3c**, ethyl acetate/acetonitrile, MnO_2_, reflux, 8 days	63
**4d**	H	OH	OH	H	**3d**, ethyl acetate/acetonitrile, MnO_2_, reflux, 24 h	55

r.t. (room temperature).

**Table 2:** Data from cyclic voltammetry, DPPH^●^ and deoxyribose degradation assays for the target compounds.

Compound	E_p_ (mV vs. ECS)	E_p/2_ (mV vs. ECS)	DPPH IC_50_ (μM)	% Inhibition of deoxyribose degradation
**3a**	688	649	n.d.	n.d.
**3b**	740	-	n.d.	n.d.
**3c**	476	361	n.d.	n.d.
**3d**	248	185	n.d.	n.d.
**3e**	113	40	3.7 ± 0.7	62.1 ± 2.3
**4a**	783	758	n.d.	n.d.
**4b**	896	712	n.d.	n.d.
**4c**	620	534	n.d.	n.d.
**4d**	254	166	12.0 ± 1.0	59.0 ± 3.5
**Trolox**	173	107	9.0 ± 0.2	23.4 ± 2.6

E_p_ (Anodic peak potential); E_p/2_ (half peak potential); SCE (saturated calomel electrode); DPPH^●^ (2,2-diphenyl-1-picrylhydrazile radical); IC_50_ (50% inhibitory concentration); n.d. (not determined).

**Table 3:** Values of minimum inhibitory concentration (MIC) on yeast and the viability of fibroblasts at the indicated concentrations of the compounds.

Compound	Antifungal Activity MIC (μM)		Cellular Viability (%)
	*Saccharomyces cerevisiae*	*Candida albicans*	Fibroblasts
**3a**	>200.0 *	>200.0 *	n.d.
**3b**	>200.0 *	>200.0 *	n.d.
**3c**	>400.0 *	>400.0 *	n.d.
**3d**	>400.0*	>400.0 *	n.d.
**3e**	**50.0**	**100.0**	**97.3 ± 1.9**
**4a**	>400.0 *	>400.0 *	n.d.
**4b**	>400.0 *	>400.0 *	n.d.
**4c**	>400.0 *	>400.0 *	n.d.
**4d**	**400.0**	**600.0**	**94.8 ± 3.5**
**Miconazole**	**100.0**	**0.78**	n.d.
**Fluconazole**	**50**	**1.56**	n.d.

n.d. (not determined). * Not achieved at highest concentration tested.
